# Survey on Discharge Support for Patients from Dementia Treatment Wards to Home and Associated Issues: A Questionnaire-Based Study in Japan

**DOI:** 10.7759/cureus.97376

**Published:** 2025-11-20

**Authors:** Chiaki Sakamoto, Seiji Nishida, Katsuma Ikeuchi, Kumiko Masuda, Yasushi Orita, Fujimaki Koichiro

**Affiliations:** 1 Department of Occupational Therapy, Faculty of Health and Welfare, Prefectural University of Hiroshima, Mihara, JPN

**Keywords:** dementia, discharge planning, discharge support, hospital, japan, questionnaires

## Abstract

Background

In recent years, the number of people with dementia and those requiring hospitalisation has continued to rise. However, several challenges remain in the provision of discharge support for this population, including inadequate collaboration between individuals and their families and inadequate support skills among care staff. This study, therefore, aimed to clarify the current state of home discharge support for individuals with dementia in specialised dementia treatment wards in Japan.

Methods

We conducted a 12-item questionnaire survey using a mixed research method targeting all 525 institutions in Japan with dementia treatment wards.

Results

Responses were received from 190 dementia treatment wards. More than 70% of people with dementia discharged home were hospitalised for more than two months. There were several barriers to discharge support, including ‘giving up life at home’, ‘inadequate support skills’, ‘regional characteristics’, ‘immature organisational culture’, and ‘constraints of family background’. In addition, groups with adequate discharge support were found to have collaborated with a wider range of professionals inside and outside the hospital compared with groups with inadequate discharge support. Discharge support teams also collaborated with people with dementia, their families, and multidisciplinary teams from the beginning of hospitalisation; experimented with different approaches to facilitate the return to home life; and implemented seamless and flexible interventions, including post-discharge follow-up, to facilitate a smooth transition back to home life.

Conclusion

This study sheds light on the practical realities of home discharge support for individuals with dementia in Japan. Although numerous challenges persist, many institutions are implementing creative and collaborative strategies to enable home discharge. The identified effective practices may serve as models, and resolving the highlighted issues is critical for further improvement.

## Introduction

As society ages, the number of people with dementia is increasing worldwide [1-3]. Currently, there are more than 55 million people with dementia worldwide, and this number is expected to grow to 139 million by 2050 [4]. The prevalence of dementia is also increasing in Japan [5]. The behavioural and psychological symptoms of dementia (BPSD) increase the care burden on the caregivers [6] and can be a major cause of hospitalisation [7]. Those with dementia are at a higher risk of experiencing a decline in physical and mental functions during hospitalisation [8] and, compared with people without dementia, are more likely to have longer hospital stays and less likely to be discharged home [9]. Additionally, because of the effects of cognitive decline on communication and understanding [10], people with dementia are more likely to be excluded from decisions about their own care and treatment [11]. Particularly regarding discharge support, people with dementia and their families are often not adequately involved in discharge planning [12-14], and effective coordination with support providers is often lacking [15]. In addition, the affected individuals’ families may lack knowledge and confidence in caregiving [16], and caregivers may lack the necessary skills to provide discharge support [17], resulting in inadequate discharge support for those with dementia.

Discharge support for people with dementia must be seamless and continuous from the early stages of hospitalisation [17] and should include collaboration between people with dementia and their families [18], provision of support to meet post-discharge needs [19], and ongoing post-discharge support [10]. The development of comprehensive, individualised support plans tailored to the needs of people with dementia by a multidisciplinary team increases the likelihood of them returning home [20]. Additionally, involving family carers in discharge support is an important factor in reducing stress and preparing for discharge to home [21].

In many psychiatric hospitals in Japan, dementia treatment wards have been established with the aim of providing short-term intensive inpatient treatment for patients with severe dementia in the acute phase who have significant BPSD [22]. These wards serve as the primary inpatient setting for patients with severe dementia and BPSD [23,24]. Dementia treatment wards provide a comprehensive support system because they are staffed by a multidisciplinary team, including psychiatrists, nurses, occupational therapists, and mental health workers [22]. Occupational therapists, in particular, play a pivotal role in supporting the daily lives of patients with dementia and facilitating their discharge [25]. However, the average length of stay in these wards is approximately 480 days, indicating prolonged hospitalisation [25], and there is a tendency for activities of daily living (ADLs) to decline because of hospitalisation [26,27]. Furthermore, discharge support is often not provided [28]. Thus, a variety of challenges exist in the provision of discharge support. Despite these problems, no previous study has specifically investigated the practices of discharge support in dementia treatment wards. Although earlier research has addressed general barriers to discharge in dementia care, no national-scale, mixed-methods study has systematically explored innovative practices and structural limitations in Japan’s dementia treatment wards. This study addresses that gap.

Therefore, the purpose of this study was to clarify the discharge support practices for returning home from dementia treatment wards in Japan, as well as the innovations, issues, and difficulties associated with these practices, using a mixed research method. Using a mixed-methods approach enables quantitative and qualitative data to be analysed together, providing new insights that would otherwise be unavailable [29]. We believe this study could help highlight good practices and innovations that might serve as models for discharge support and elucidate issues and difficulties that could lead to the identification of problems and the proposal of solutions. Given the increase in the number of people with dementia worldwide and the expected increase in hospital admissions, it is important to clarify current practices and issues related to home discharge support for people with dementia.

## Materials and methods

Study design

A mixed-methods research design was used to conduct a web-based questionnaire survey. To gain a deeper understanding of discharge support practices in dementia treatment wards, we used a convergent mixed method [29] to collect and compare quantitative and qualitative data. The study is reported in line with the Mixed Methods Reporting in Rehabilitation & Health Sciences (MMR‐RHS) guidelines [30].

Study subjects

This study included all 525 dementia treatment wards registered with the Regional Bureau of Health, Labour and Welfare as of September 2022.

Survey methods and procedures

Survey Creation

First, a questionnaire draft was developed based on the literature on home discharge support in Japanese dementia treatment wards [27,28,31,32]. The authors, who have experience in discharge support for people with dementia, reviewed the content of the questionnaire to ensure that the respondents were not unduly burdened, and that the intent of the questions was clear and expected responses could be obtained.

The questionnaire was developed for the purpose of this survey and was not intended as a psychometric scale. Therefore, statistical examination of reliability and construct validity was not conducted. Instead, content validity was ensured through expert review and pilot testing. Specifically, to enhance content validity, the questionnaire items were reviewed and refined through multiple discussions between the authors. The clarity and feasibility of the questions were confirmed through pilot testing with two occupational therapists.

The final questionnaire contained the following 12 items (Appendix): 1) Item one: Respondents’ profession; 2) Item two: Respondents’ perception of discharge support (1 point: never adequate - 6 points: very adequate); 3) Item three: Selection of persons or institutions involved in discharge support by using a multiple-choice method; 4) Item four: Number of beds in the main dementia treatment wards; 5) Item five: Number of patients admitted from home in the past year; 6) Item six: Number of patients discharged to home among those in item five; 7) Item seven: Average length of hospital stay for those falling under item six; 8) Item eight: Discharge support practices before and at the time of admission; 9) Item nine: Discharge support practices during hospitalisation; 10) Item 10: Discharge support practices at the time of discharge and after discharge; 11) Item 11: Innovations in discharge support; 12) Item 12: Issues or difficulties in discharge support.

To reduce respondent burden, items five to seven were pre-coded. For items eight to 12, which were open-ended questions, examples were provided below each question to help respondents answer. The questionnaire was created using Google Forms (Google, California, US), and data were collected online. Personal information, such as email addresses, was not collected. In this survey, discharge support was defined as support provided to enable people with dementia to return to their homes in familiar neighbourhoods, excluding shared accommodations.

Request for Study Participation

A document requesting study participation was prepared using a single sheet of A4 paper. It included an explanation of the study, a request for cooperation, a QR code and URL to access the questionnaire, information about informed consent and the handling of personal information, and contact details for any questions about the study. The document was sent to all 525 dementia treatment wards.

Those who agreed to participate completed the questionnaire, one per hospital organisation. The questionnaire was to be completed by occupational therapists or rehabilitation professionals working exclusively in dementia treatment wards or those who were considered suitable to complete the survey by the occupational therapists themselves. This is because they are directly involved in supporting patients’ engagement in daily activities and facilitating transitions from hospital to home [25]. Their perspectives were essential to understanding current practices and challenges related to home discharge support for people with dementia.

Survey period

The survey period was set for approximately two months, from 1 November 2022 to 6 January 2023. If the response rate was less than 20% by 15 December 2022, the questionnaire was resent to the survey institutions to improve the response rate.

Analysis method

Descriptive statistics were calculated for items one, four, five, six and seven. Item two was divided into two groups: the ‘adequate discharge support group’ (options: very adequate, adequate, and somewhat adequate) and the ‘inadequate discharge support group’ (options: not very adequate, not adequate, and never adequate). The proportions of individuals and organisations involved in discharge support in item three were compared between the two groups using the chi-squared test or Fisher’s exact probability test (P<0.05).

In addition, the practices in items eight, nine, and 10; innovations in item 11; and issues and difficulties pertaining to item 12 were analysed objectively and systematically based on the responses provided by the survey participants. This analysis was conducted with reference to Berelson’s content analysis [33,34] to clarify more specific details. All descriptive data were divided into record units. Then, record units with consistent expressions and meanings were organised into the same record unit group. Subsequently, these were classified into subcategories and categories based on similarities and differences and given suitable names. The total number of units in each category and their respective percentages were then calculated. This analysis was conducted under the guidance of co-authors with experience in content analysis research. In addition, the co-authors conducted member checks to assess validity. Finally, the reliability of the generated categories was confirmed. One co-author and one occupational therapist with experience in discharge support for people with dementia in dementia treatment wards or psychiatric wards reclassified the categories in the extracted records and calculated Cohen’s κ coefficient [35] for the consistency rate. Record units were extracted as reported previously [34], and 15% of the record units were selected randomly. The criteria used to determine the reliability of the categories in the analysis were an agreement rate of ≥70% [34] and a Cohen’s κ coefficient of ≥0.61 [35].

IBM SPSS Statistics for Windows, Version 28 (Released 2021; IBM Corp., Armonk, New York, United States).

Ethical considerations

This study was conducted in accordance with the principles of the Declaration of Helsinki and was approved by the Research Ethics Committee of the Prefectural University of Hiroshima (approval number: 22MH027-01). The study information sheet and Google Form clearly explained the purpose of the research, emphasised that participation was voluntary, and assured participants that refusal or withdrawal would not result in any disadvantage. It was also stated that all data would be anonymised to prevent the identification of individuals during data processing and publication. Additionally, the name, affiliation, and contact information of the Principal Investigator were provided for inquiries, and participants were informed that the researchers would be available to respond to any questions.

## Results

The response rate to the questionnaire was 34.3%, with responses received from 190 of the 525 institutions. The respondents included 187 occupational therapists (98.4%) and three mental health workers (1.6%).

The number of beds in the main dementia treatment wards of the participating institutions was as follows: 60 beds (25.3%), 50 beds (17.9%), and 48 beds (5.8%).

With regard to the number of hospital admissions from home in the past year, the responses were as follows: none, six respondents (3.2%); 1-20, 129 respondents (67.9%); 21-40, 41 respondents (21.6%); 41-60, six respondents (3.2%); and ≥61, eight respondents (4.2%). Of these, regarding the number of home discharges, the responses received were as follows: none, 45 respondents (23.7%); 1-20, 141 respondents (74.2%); 21-40, six respondents (2.2%); and 41-60 or ≥61, no respondents (0%). With regard to the average length of hospital stay for patients admitted from home and discharged to home in the past year, the responses received were as follows: 1-30 days, 13 respondents (6.9%); 31-60 days, 28 respondents (17.2%); 61-90 days, 48 respondents (31.7%); and ≥91 days, 101 respondents (44.2%).

The responses regarding the adequacy of discharge support were as follows: ‘very adequate’, two responses; ‘adequate’, 10 responses; ‘somewhat adequate’, 31 responses; ‘not very adequate’, 87 responses; ‘not adequate’, 34 responses; and ‘never adequate’, 26 responses. When the responses were divided into the adequate and inadequate discharge support groups, the two groups comprised 43 (22.6%) and 147 (77.4%) cases, respectively.

The results of the comparison of the types of professions and institutions involved in home discharge support between the adequate and inadequate discharge support groups are shown in Table 1.

**Table 1:** Professions and organisations involved in discharge support This table shows the results of a comparison of the types of professions and organisations involved in home discharge support between the groups with adequate and inadequate discharge support. The professions and organisations involved were differentiated between those inside and outside the hospital. *P<0.05; **P<0.001; ^†^Dietitian, Speech-language-hearing therapist, Centre counsellor, Nursing assistant, Pharmacist; ^‡^Municipal Welfare Department, Public Health Centre, Guardian, Daily life care in communal living for elderly people with dementia, Short-term stay at a care facility (short stay).

Related professions and organisations		Adequate discharge support group, n=43; n (%)	Inadequate discharge support group, n=147; n (%)	P-value	
In hospital	The person with dementia	29 (67.4)	83 (56.5)	0.198	
	Family members and primary carer in the lives of people with dementia	37 (86.0)	140 (95.2)	0.078	
	Doctor	43 (100.0)	140 (95.2)	0.353	
	Nurse	43 (100.0)	140 (95.2)	0.353	
	Carer/Carer’s assistant	30 (69.8)	51 (34.7)	<0.001	**
	Physical therapist	9 (20.9)	26 (17.7)	0.629	
	Occupational therapist	39 (90.7)	120 (81.6)	0.157	
	Certified psychologist	18 (41.9)	33 (22.4)	0.012	*
	Psychiatric social worker	43 (100.0)	141 (95.9)	0.340	
	Other^†^	6 (14.0)	8 (5.4)	0.091	
Out of hospital	Care manager	41 (95.3)	124 (84.4)	0.061	
	Community comprehensive support centre	33 (76.7)	83 (56.5)	0.016	*
	Home care office	30 (69.8)	52 (35.4)	<0.001	**
	Commuting for care (day service)	29 (67.4)	64 (43.5)	0.006	**
	Commuting rehabilitation service (day care)	21 (48.8)	41 (27.9)	0.010	*
	Commuting care for elderly people with dementia/Dementia day care	19 (44.2)	37 (25.2)	0.016	*
	Psychiatric day care	8 (18.6)	24 (16.3)	0.726	
	Home-visit care (home help service)	24 (55.8)	41 (27.9)	<0.001	**
	Home-visit nursing care	28 (65.1)	47 (32.0)	<0.001	**
	Psychiatric home-visit nursing care	16 (37.2)	28 (19.0)	0.013	*
	Minseiiin (commissioned welfare volunteer)	3 (7.0)	16 (10.9)	0.573	
	Neighbours	2 (4.7)	9 (6.1)	1.000	
	Friends and other people close to the person with dementia	7 (16.3)	8 (5.4)	0.047	*
	Other^‡^	1 (2.3)	3 (2.0)	1.000	

Within the hospital, significant differences were observed for care/caregiver assistants (P<0.001) and certified psychologists (P=0.012). Outside the hospital, community comprehensive support centre (P=0.016), home care office (P<0.001), commuting for care (day service) (P=0.006), commuting rehabilitation service (day care) (P=0.010), commuting care for elderly people with dementia/dementia day care (P=0.016), home-visit care (home help service) (P<0.001), home-visit nursing care (P<0.001), psychiatric home-visit nursing care (P=0.013), and friends and other people close to the person with dementia (P=0.047) showed significant differences.

The results of the analysis of home discharge support practices are presented in Table 2.

**Table 2:** Practices of home discharge support for each period Note. This table shows the results of the analysis of home discharge support practices in dementia treatment wards in Japan. This content analysis was conducted with reference to Berelson’s content analysis [33,34]. The record units were organised into the same group, classified into subcategories and categories based on similarities and differences, and given appropriate names. Furthermore, the total number of units within each category and their respective proportions were calculated.

Period	Total number of record units	Categories	Number of record units (percentage)	Subcategories
Before and at the time of hospitalisation	242	Reviewing the current situation	155 (64.0%)	Understanding the patient’s life situation before hospital admission
				Confirming the wishes and preferences of the person and their family
				Sharing information between stakeholders
		Planning and initiating discharge support	30 (12.4%)	Sharing information between different professions and setting discharge targets
				Preparing for life in the hospital
				Individual assessment and development of a support plan
		Assessing and adjusting the home environment	13 (5.4%)	Understanding the home environment
				Service adjustment
		No support	44 (18.2%)	No support
During hospitalisation	291	Organising information and unifying support	138 (47.4%)	Information sharing and policy confirmation between different professions
				Providing care based on the home environment
		Collaborating with the family	28 (9.6%)	Considering discharge from the hospital to home with family
				Providing information to the family
		Implementing rehabilitation	26 (8.9%)	Assessment and training for activities of daily living and physical and mental functions
				Incorporating rehabilitation into everyday life
		Challenges in home life	68 (23.4%)	Trial and adjustment of home-based services
				Confirmation of adaptation at home
		No support	31 (10.7%)	No support
At the time of discharge and after discharge	228	Sharing information about what is needed to live at home	101 (44.3%)	Handover for home life through interprofessional conferences
				Handover to regional supporters
				Providing information and care guidance to families
		Preparing for life at home	39 (17.1%)	Reviewing adaptation at home and home services
				Strengthening links with local social resources
		Following up after discharge	15 (6.6%)	Checking living conditions through visits
				Checking the living situation at the time of the outpatient visit
		Do not know	3 (1.3%)	Do not know
		No support	70 (30.7%)	No support

A total of 242 record units were analysed for the periods before and at the time of hospitalisation. These records were divided into 16 groups of aggregated record units, which were further divided into nine subcategories based on the similarity of meaning and then into four categories. For the period of hospitalisation, 291 record units were analysed. These were divided into 24 groups of aggregated record units. These were further organised based on the similarity of meaning, resulting in nine subcategories and five categories. In total, 228 records were analysed for the discharge and post-discharge periods. They were then divided into 15 groups of aggregated record units. Further organisation based on the similarity of meaning resulted in the generation of nine subcategories and five categories.

A total of 231 record units were analysed for innovations in home discharge support. These were divided into 38 groups of aggregated record units. In addition, 19 subcategories and eight categories were created based on similarities in meaning. The results of this analysis are shown in Table 3.

**Table 3:** Innovations for home discharge support This table shows the results of the analysis of innovations for home discharge support in dementia treatment wards in Japan. This content analysis was conducted with reference to Berelson’s content analysis [33,34]. The record units were organised into the same group, classified into subcategories and categories based on similarities and differences, and given appropriate names. Furthermore, the total number of units within each category and their respective proportions were calculated.

Total number of record units	Categories	Number of record units (percentage)	Subcategories
231	Respect for the individual	7 (3.0%)	Confirming the patient’s wishes
			Creating an inpatient life that suits each individual
	Ensuring ability to live at home	38 (16.5%)	Maintaining and practising activities of daily living and physical and mental functions
			Rehabilitation and care for home life
	Adjusting to home life	23 (10.0%)	Assessing the home environment and intervening
			Trials of outings and overnight stays
			Proposing and coordinating social resources in the area
	Approaching the family	36 (15.6%)	Confirmation of the family’s wishes
			Regular contact and hospital visits
			Sharing information with the family
			Assessment of the ability of family members to provide care and caregiving advice
	Creating hospital systems	17 (7.4%)	Unifying the direction of multidisciplinary collaboration
			Utilizing medical fees
	Sharing with the community	15 (6.5%)	Collaborating with local supporters
			Sharing information with family and supporters
			Post-discharge interviews and support
	Flexible response to infectious diseases	6 (2.6%)	Practising alternative methods during the coronavirus disease 2019 pandemic
	No innovation	89 (38.5%)	No innovation

A total of 277 record units were analysed for issues and difficulties pertaining to home discharge support. They were then divided into 90 groups of aggregated record units. In addition, 37 subcategories and 13 categories were created based on similarities in meaning. The results of this analysis are shown in Table 4.

**Table 4:** Issues and difficulties pertaining to home discharge support This table shows the results of the analysis of the issues and difficulties pertaining to home discharge support in dementia treatment wards in Japan. This content analysis was conducted with reference to Berelson’s content analysis [33,34]. The record units were organised into the same group, classified into subcategories and categories based on similarities and differences, and given appropriate names. Furthermore, the total number of units within each category and their respective proportions were calculated.

Total number of record units	Categories	Number of record units (percentage)	Subcategories
277	Mismatch of vision and intent	15 (5.4%)	Difference in perceptions or desires
			Disagreements between the person, their family, and supporters
	Constraints of family background	68 (24.5%)	The limits of family members’ ability to provide care
			Difficulties in gaining family understanding and cooperation
			Absence of key persons owing to weak family relationships
			Caregiver fatigue
			Poor relationship with family
			Discharge refused by family
	Financial burden	5 (1.8%)	Limited choice owing to financial constraints
			Financial problems
	Giving up life at home	33 (11.9%)	Wishes to be in an institution from the time of hospital admission
			No one goes home
	Immature organisational culture	24 (8.7%)	Hospitals are not in the habit of trying to get patients discharged home
			The hospital does not have a discharge support system
			Staff have a low level of awareness of discharge support
			Institutionalisation in the hospital
	Inadequate organisational systems	42 (15.2%)	Difficulty in finding time for individual support
			Inadequate support owing to lack of manpower
			Undue burden on supporters
			Lack of coordination between the family and hospital
			Lack of multidisciplinary collaboration and information sharing
			Bias in the types of professions involved in discharge support
			Lack of support for families
	Systemic barriers	2 (0.7%)	Home visits are difficult owing to medical fees
	Inadequate support skills	16 (5.8%)	Inadequate experience and knowledge on the part of supporters in helping patients discharge from the hospital
			Repeated hospital admissions/discharges
	Regional characteristics	12 (4.3%)	Inadequate available social resources
			Habit of hiding dementia
			Ageing of the whole region
			Poor location of the hospital
	High level of care required	11 (4.0%)	Physical and mental functions require constant care
			Severe symptoms of dementia
	Negative effects of hospitalisation	10 (3.6%)	Decline in activities of daily living and progression of dementia after hospitalisation
			Prolonged hospital stay
	Barriers caused by the COVID-19 pandemic	17 (6.1%)	COVID-19 pandemic restrictions on cooperation and collaboration with the family
			COVID-19 pandemic restrictions on face-to-face assessment and support
	No issues or difficulties	22 (7.9%)	No issues or difficulties

Subsequently, an evaluation was conducted to ascertain the reliability of the categories. We calculated the agreement rate for the categorisation in 15% of the randomly selected datasets using Cohen’s κ coefficient. The results were as follows (co-author and other): discharge support practices before and at hospitalisation (item eight), κ=0.851 and κ=0.950, respectively; discharge support practices during hospitalisation (item nine), κ=0.791 and κ=0.986; discharge support practices at and after discharge (item 10), κ=0.892 and κ=0.965; innovations for home discharge support (item 11), κ=0.913 and κ=0.926, respectively; and issues or difficulties pertaining to home discharge support (item 12), κ=0.798 and κ=0.875, respectively. These κ coefficients were classified as ranging from ‘almost perfect agreement’ to ‘substantial agreement’ [35].

## Discussion

This study examined the current status, practices, and challenges of home discharge support for people with dementia in dementia treatment wards in Japan, integrating both quantitative and qualitative findings.

Most respondents were occupational therapists, reflecting the survey’s focus on professionals directly involved in discharge support for people with dementia. As expected, their responses provided valuable insights into the current situation and challenges related to supporting patients with dementia in returning home from treatment wards.

The quantitative results indicated that home discharge rates remained low despite relatively long hospital stays and that perceptions of the adequacy of discharge support varied. These findings suggest that, although discharge support is being implemented, its effectiveness may be limited by organizational and contextual factors.

The qualitative analysis supported this interpretation by identifying barriers such as limited interprofessional coordination, insufficient community resources, and challenges in maintaining daily functioning after discharge, alongside innovative approaches such as early occupational therapy involvement and collaboration with community services.

Overall, these findings indicate that the adequacy of discharge support depends not only on individual professional efforts but also on systemic factors such as teamwork and continuity of care. The following sections discuss these results in relation to previous literature and their implications for improving discharge support practices.

Current home discharge status and discharge support issues and difficulties

Approximately 70% of respondents reported that the number of hospitalisations from home in the past year was ‘1-20’, indicating a low number of hospitalisations from home. In addition, approximately 25% of these respondents stated that none of the patients hospitalised from home were discharged home. Furthermore, in terms of issues and difficulties pertaining to discharge support, categories such as ‘mismatch of vision and intent’, ‘giving up life at home’, ‘immature organisational culture’, and ‘negative effects of hospitalisation’ were identified. These findings are consistent with those of previous studies that identified hospitalisation-related declines in ADL and functional ability [8,20,21], inadequate discharge support [10,22], severe BPSD, and difficulties in the medical management of dementia care [7,26,36] as factors hindering discharge to home. In addition, previous research [26] reported that over 60% of people admitted from home were unable to return home, highlighting the challenge in achieving discharge from dementia treatment wards to the home environment.

In 45% of cases, the average length of hospital stay for patients discharged home from the hospital was approximately three months or more. Although this is shorter than that in previous studies [25], the Ministry of Health, Labour and Welfare aims for discharge within two months [37]; hence, there is room for improvement. Issues identified in previous studies, such as inadequate coordination between patients with dementia and their families [12-15], a lack of collaboration [16,18,19], insufficient support skills among caregivers [18], and a lack of resources and manpower [38], are consistent with the categories of issues and difficulties pertaining to discharge support, including ‘constraints of family background’, ‘inadequate organisational culture’, ‘inadequate support skills’, and ‘high level of care required’. Based on the results of this survey, these issues may contribute to a prolonged hospital stay. Thus, there are multiple issues in supporting people with dementia on discharge, not only related to the condition of the person with dementia but also involving families, support providers, and organisational systems.

Additionally, this study identified new issues and difficulties related to discharge support, categorised as ‘financial burden’, ‘systemic barriers’, ‘regional characteristics’, and ‘barriers caused by the COVID-19 pandemic’. ‘Financial burden’ was found to influence discharge options, such as transfer to a nursing home, whereas ‘systemic barriers’ included limitations such as the inability to conduct home visits owing to problems with the medical reimbursement system. In addition, ‘regional characteristics’ were identified as barriers to discharging people with dementia home, including the practice of hiding dementia and a lack of community resources. It should be noted that this survey was conducted during the COVID-19 pandemic, which led to the creation of the category ‘barriers caused by the COVID-19 pandemic’. The pandemic resulted in severe restrictions on visits and outings at the time of the survey, making it difficult to provide support through outreach. Presently, certain facilities persist in implementing restrictions on visits and outings. Consequently, it is hoped that alternative means of support will be explored. These results highlight the various issues and difficulties pertaining to home discharge support in dementia treatment wards in Japan.

Adequacy of discharge support and multidisciplinary collaboration

The adequate discharge support group collaborated significantly more with various professionals and institutions (especially outside the hospital) for discharge support compared with the inadequate discharge support group. Although this study did not clarify the causal relationship between better support and collaboration with different professionals, it suggests that multidisciplinary collaboration may improve the quality of discharge support provided by the support staff. Dementia care is a demanding profession with high levels of stress [39], and interdisciplinary teamwork is recommended [40]. Furthermore, multidisciplinary collaboration is associated with higher job satisfaction [41] and intention to continue working [42]. Thus, the practice of multidisciplinary collaboration in the care of people with dementia is strongly supported.

Practical support and innovative approaches pertaining to discharge to home

Regarding the practice of discharge support, various record units were extracted. Before and at hospitalisation, the practices pertaining to discharge support included ‘reviewing the current situation’, such as prehospital living conditions and intentions; ‘planning and initiating discharge support’ by a multidisciplinary team based on related information; and ‘assessing and adjusting the home environment’. During hospitalisation, a multidisciplinary team implemented ‘organising information and unifying support’ based on the assumption of home life, ‘collaborating with the family’, and ‘implementing rehabilitation’ including ADL assessment and training, as well as addressed ‘challenges in home life’ such as trial use of home services and confirmation of home adaptation. At and after discharge, the following practices were implemented: ‘sharing information about what is needed to live at home’ with community support providers; ‘preparing for life at home’, including confirmation of home adaptation and services; and ‘following up after discharge’, including visits or outpatient visits to confirm living conditions. Early discharge support from the start of hospitalisation is effective in shortening the hospital stay [43], and the practice of discharge support from the time of admission, which was also evident in this study, is considered effective for discharge to home.

In terms of innovations in discharge support, this study, similar to previous ones, highlighted the importance of collaborative approaches that respect the wishes of people with dementia and their families [10,11], as well as the involvement of family caregivers in discharge support [12]. In addition, the categories ‘ensuring ability to live at home’ (e.g., maintaining ADLs), ‘adjusting to home life’ (e.g., trying overnight stays outside the home), ‘creating hospital systems’ (e.g., multi-professional collaboration), and ‘sharing with the community’ (e.g., collaboration with community support providers) were generated. These findings indicate that supporters use various creative approaches in their efforts to support discharge.

This study has shown that seamless discharge support is being considered in dementia treatment wards, which face a variety of issues and difficulties, by focusing on home life from the time of admission; collaborating with patients with dementia, their families, and various professionals to make trial-and-error attempts to return home; and providing follow-up care even after discharge. However, some wards reported that they did not implement discharge support at any stage. Because the results of this survey are a compilation of practices from several hospitals, there is likely variability in the implementation of discharge support across hospitals, and it cannot be concluded that all wards provide adequate discharge support. Nevertheless, the practices and innovations pertaining to discharge support identified in this study may serve as valuable references for improving discharge support in dementia treatment wards facing the issues highlighted in previous studies [10-16,18,25-28,38]. 

Limitations of the study

First, responses were collected only from occupational therapists working exclusively in dementia treatment wards or individuals designated by them. Other professional or institutional characteristics, including years of experience in discharge support or tenure at the facility, were not obtained, which may have influenced the findings. Second, the adequacy of discharge support was based on respondents’ subjective perspectives, which may have introduced response bias. Third, the study was limited to institutions that responded to the questionnaire, and practices in non-responding institutions remain unknown, raising the possibility of selection bias.

In addition, although the study employed a mixed-methods design with systematic content analysis, qualitative categorisation may still involve interpretive subjectivity, even with confirmed inter-rater reliability. Finally, given that all the participating institutions were located in Japan, the generalisability of the findings to other countries or healthcare systems may be limited.

## Conclusions

This study examined current practices for supporting home discharge in dementia treatment wards across Japan. Despite numerous challenges, many institutions are making continuous efforts to enable discharge through a range of strategies. With the projected increase in the number of people with dementia, and consequently hospitalisations, home discharge will become an increasingly important aspect of care. The effective practices identified in this study may serve as valuable models, and addressing the issues highlighted will be essential to help individuals with dementia continue living in their communities.

## Appendices

Survey questionnaire on support for discharge home from dementia treatment wards in Japan

Item 1

Please select your job. If you select 'Other', please specify your job.

¨  Doctor

¨  Nurse

¨  Carer/Carer’s assistant

¨  Physical therapist

¨  Occupational therapist

¨  Certified psychologist

¨  Psychiatric social worker

¨  Other

Item 2

Please select the level of support provided for discharge home from the dementia treatment ward.

6. Very adequate

5. Adequate

4. Somewhat adequate

3. Not very adequate

2. Not adequate

1. Never adequate

Item 3

1. In hospital: Please select all individuals who are involved in supporting the discharge of patients with dementia to their homes. If you select 'Other', please provide details of the relevant individual.

¨ The person with dementia

¨  Family members and key persons in the lives of people with dementia

¨  Doctor

¨  Nurse

¨  Carer/Carer's assistant

¨  Physical therapist

¨  Occupational therapist

¨  Certified psychologist

¨  Psychiatric social worker

¨  Other

2. Out of hospital: Please select all the relevant parties and facilities outside the hospital that are involved in supporting the discharge of dementia patients to their homes. If you select 'Other', please provide details of the relevant individual.

¨  Care manager

¨  Community comprehensive support centre

¨  Home care office

¨  Commuting for care (day service)

¨  Commuting rehabilitation service (day care)

¨  Commuting care for elderly people with dementia/Dementia day care

¨  Psychiatric day care

¨  Home-visit care (home help service)

¨  Home-visit nursing care

¨  Psychiatric home-visit nursing care

¨  Minseiiin (commissioned welfare volunteer)

¨  Neighbours

¨  Friends and other people close to the person with dementia

¨  Other

Item 4

Please specify the number of beds in the dementia treatment ward.

Item 5

Please select the option that corresponds to the number of people admitted to hospital from their own homes during the past year (1 September 2021 to 30 September 2022). In the context of this survey, residential care facilities such as nursing homes, serviced housing for the elderly, and dementia-specific group homes are not classified as "home".

¨ No admissions from home

¨  1-20 persons

¨  21-40 persons

¨  41-60 persons

¨  61 persons or more

Item 6

Please select the number of individuals from Item 5 who were discharged to their own homes.

¨  No patients discharged to their home

¨  1-20 persons

¨  21-40 persons

¨  41-60 persons

¨  61 persons or more

Item 7

Please select the option that corresponds to the mean duration of hospitalisation for Item 6.

¨  1-30 days

¨  31-60 days

¨  61-90 days

¨  91 days or more

Item 8

What support is provided before and at hospitalisation to facilitate discharge back to the patient’s home? Please provide a detailed description of the support provided, including the following points: timeframe, location, provider, recipient, actions, etc.

Example 1: Upon admission, a psychiatric social worker interviews the family about the patient's living situation prior to hospitalisation.

Example 2: Several days after admission, a nurse and occupational therapist visit the patient's home. Together with the dementia patient, their family, and the care manager, they review the patient's condition prior to admission and clarify specific discharge goals.

Item 9

What support is provided during hospitalisation to facilitate discharge to the patient’s home? Please provide a detailed description of the support provided, including the following points: timeframe, location, provider, recipient, actions, etc.

Example 1: Hold a multidisciplinary conference early in the hospital stay to share discharge goals.

Example 2: When behavioural and psychological symptoms of dementia have stabilised, take the dementia patient out to experience the day service they will use after discharge.

Item 10

What support is provided at and after discharge to facilitate discharge back to the patient's home? Please provide a detailed description of the support provided, including the following points: timeframe, location, provider, recipient, actions, etc.

Example 1: A pre-discharge care conference is held with the dementia patient, their family, the doctors, nurses, occupational therapists, psychiatric social workers, care managers, and carers to arrange the transition to home life.

Example 2: Following discharge, a home visit is conducted to assess living conditions, with information shared with the staff who provided support during the hospital stay.

Item 11

Please provide a description of the innovations you are implementing to support patients returning home. Provide as much detail as possible.

Item 12

Please provide a description of any difficulties or issues you experience related to home discharge support for patients with dementia. Provide as much detail as possible.

## References

[1] Zhong S, Xiao C, Li R (2025). The global, regional, and national burdens of dementia in 204 countries and territories from 1990 to 2021: a trend analysis based on the Global Burden of Disease Study 2021. Medicine (Baltimore).

[2] Cao Q, Tan CC, Xu W (2020). The prevalence of dementia: a systematic review and meta-analysis. J Alzheimers Dis.

[3] Fiest KM, Jetté N, Roberts JI (2016). The prevalence and incidence of dementia: a systematic review and meta-analysis. Can J Neurol Sci.

[4] (2025). Dementia fact sheet. https://www.alzint.org/resource/dementia-fact-sheet/.

[5] Okamura H, Ishii S, Ishii T, Eboshida A (2013). Prevalence of dementia in Japan: a systematic review. Dement Geriatr Cogn Disord.

[6] de Vugt ME, Stevens F, Aalten P (2004). Do caregiver management strategies influence patient behaviour in dementia?. Int J Geriatr Psychiatry.

[7] Quante A, Sulejmani A (2017). Prevalence and pharmacotherapy of behavioral and psychological symptoms of dementia in a geriatric psychiatry unit: a retrospective analysis. Prim Care Companion CNS Disord.

[8] Hoogerduijn JG, Grobbee DE, Schuurmans MJ (2014). Prevention of functional decline in older hospitalized patients: nurses should play a key role in safe and adequate care. Int J Nurs Pract.

[9] Amjad H, Sekhon VK, Wolff JL, Samus QM, Roth DL (2025). Hospitalization outcomes among older adults living undiagnosed or unaware of dementia. Alzheimers Dement (Amst).

[10] Bauer M, Fitzgerald L, Haesler E, Manfrin M (2009). Hospital discharge planning for frail older people and their family. Are we delivering best practice? A review of the evidence. J Clin Nurs.

[11] Dupuis SL, Gillies J, Carson J, Whyte C, Genoe R, Loiselle L, Sadler L (2012). Moving beyond patient and client approaches: mobilizing ' authentic partnerships ' in dementia care, support and services. Dementia.

[12] Chenoweth L, Kable A, Pond D (2015). Research in hospital discharge procedures addresses gaps in care continuity in the community, but leaves gaping holes for people with dementia: a review of the literature. Australas J Ageing.

[13] Mockford C (2015). A review of family carers' experiences of hospital discharge for people with dementia, and the rationale for involving service users in health research. J Healthc Leadersh.

[14] Popejoy LL, Moylan K, Galambos C (2009). A review of discharge planning research of older adults 1990-2008. West J Nurs Res.

[15] Fitzgerald LR, Bauer M, Koch SH, King SJ (2011). Hospital discharge: recommendations for performance improvement for family carers of people with dementia. Aust Health Rev.

[16] Galvin JE, Kuntemeier B, Al-Hammadi N, Germino J, Murphy-White M, McGillick J (2010). "Dementia-friendly hospitals: care not crisis": an educational program designed to improve the care of the hospitalized patient with dementia. Alzheimer Dis Assoc Disord.

[17] Jamieson M, Grealish L, Brown JA, Draper B (2016). Carers: The navigators of the maze of care for people with dementia-a qualitative study. Dementia (London).

[18] Stockwell-Smith G, Moyle W, Marshall AP (2018). Hospital discharge processes involving older adults living with dementia: an integrated literature review. J Clin Nurs.

[19] Gustavson AM, Hudson EM, Wisdom JP (2025). Identifying patient, care partner, and clinician needs for functional recovery following hospitalization when dementia is present. J Am Med Dir Assoc.

[20] Ellis G, Gardner M, Tsiachristas A (2017). Comprehensive geriatric assessment for older adults admitted to hospital. Cochrane Database Syst Rev.

[21] Pritchard E, Cussen A, Delafosse V, Swift M, Jolliffe L, Yeates H (2020). Interventions supporting caregiver readiness when caring for patients with dementia following discharge home: a mixed-methods systematic review. Australas J Ageing.

[22] Tamai A (2016). BPSD from the perspective of the new orange plan (Article in Japanese). Seishin Shinkeigaku Zasshi.

[23] Yoshimura A, Lebowitz A, Bun S, Aiba M, Ikejima C, Asada T (2016). A comparative analysis of dementia inpatient characteristics: results from a nationwide survey of different care facilities in Japan. Psychogeriatrics.

[24] Ono T, Tamai A, Takeuchi D, Tamai Y (2014). Factors related to day-care clinic outcomes for dementia patients: differences between hospitalization in the dementia ward and institutionalization. Psychogeriatrics.

[25] Morikawa T, Maeda K, Osaki T, Kajita H, Yotsumoto K, Kawamata T (2017). Admission of people with dementia to psychiatric hospitals in Japan: factors that can shorten their hospitalizations. Psychogeriatrics.

[26] Matsubara S (2012). The function of psychiatric hospitals in the treatment of dementia. Psychogeriatrics.

[27] Osaki T, Maeda K (2014). A questionnaire survey on dementia care wards: examining factors contributing to shortening hospital stays (Article in Japanese). J Geriatr Psychiatry.

[28] Okamura H, Ishii S, Ishii T, Fuchino K (2025). A nationwide survey on the hospitalization of patients with dementia admitted to psychiatric hospitals due to BPSD (Article in Japanese). J Jpn Assoc Psychiatr Hosp.

[29] Creswell JW, Creswell JD (2009). Research Design: Qualitative, Quantitative, and Mixed Methods Approaches. https://www.ucg.ac.me/skladiste/blog_609332/objava_105202/fajlovi/Creswell.pdf.

[30] Tovin MM, Wormley ME (2023). Systematic development of standards for mixed methods reporting in rehabilitation health sciences research. Phys Ther.

[31] Yuasa M, Ogawa T, Ishizuka A, Uchimura J, Honda J, Takei T (2007). Factors in long-term hospitalization and discharge support in special care units for the demented elderly: group interviews with nurses (Article in Japanese). J Health Care Nurs.

[32] Suzuki Y (2019). Factors at admission that affect home discharge in dementia wards for patients with Alzheimer's disease (Article in Japanese). J Jpn Soc Dementia Care.

[33] Albig W (1952). Berelson, Bernard. Content analysis in communication research. Ann Am Acad Pol Soc Sci.

[34] Funashima N (2007). The Challenge of Qualitative Research, 2nd Edition (Book in Japanese). https://www.igaku-shoin.co.jp/book/detail/27253#:~:text=%E6%9B%B4%E6%96%B0%E6%83%85%E5%A0%B1%20*%20%E5%BA%8F%E6%96%87%20*%20%E7%9B%AE%E6%AC%A1.

[35] Landis JR, Koch GG (1977). The measurement of observer agreement for categorical data. Biometrics.

[36] Merche J, Van Durme T, Sibille FX, Buret L, Schoevaerdts D, De Brauwer I, de Saint-Hubert M (2025). Geriatric assessment in Belgian nursing homes: qualitative insights. BMC Health Serv Res.

[37] (2025). Study Team for Building a New Community Mental Health Care System (Article in Japanese). https://www.mhlw.go.jp/stf/houdou/2r9852000001whvj.html.

[38] Ibekaku MC, Ripley S, Alizadehsaravi N (2024). Barriers and facilitators to providing rehabilitation for long-term care residents with dementia: a qualitative study. BMC Geriatr.

[39] Edvardsson D, Sandman PO, Nay R, Karlsson S (2009). Predictors of job strain in residential dementia care nursing staff. J Nurs Manag.

[40] Maki Y, Sakurai T, Okochi J, Yamaguchi H, Toba K (2018). Rehabilitation to live better with dementia. Geriatr Gerontol Int.

[41] Peter KA, Voirol C, Kunz S, Gurtner A, Renggli F, Juvet T, Golz C (2024). Factors associated with health professionals' stress reactions, job satisfaction, intention to leave and health-related outcomes in acute care, rehabilitation and psychiatric hospitals, nursing homes and home care organisations. BMC Health Serv Res.

[42] Heijkants CH, De Wind A, Van Hooff ML, Geurts SA, Boot CR (2024). Sustainable employability of long-term care staff in self-managing teams: a qualitative study. J Adv Nurs.

[43] Sanatinia R, Burns A, Crome P (2021). Factors associated with shorter length of admission among people with dementia in England and Wales: retrospective cohort study. BMJ Open.

